# From Parametric Representation to Dynamical System: Shifting Views of the Motor Cortex in Motor Control

**DOI:** 10.1007/s12264-022-00832-x

**Published:** 2022-03-17

**Authors:** Tianwei Wang, Yun Chen, He Cui

**Affiliations:** 1 Center for Excellence in Brain Science and Intelligent Technology, Institute of Neuroscience, Chinese Academy of Sciences, Shanghai, 200031 China; 2 Shanghai Center for Brain and Brain-inspired Intelligence Technology, Shanghai, 200031 China; 3 University of Chinese Academy of Sciences, Beijing, 100049 China

**Keywords:** Dimensionality reduction, Neural network, Machine learning, Population decoding, Brain-machine interface

## Abstract

In contrast to traditional representational perspectives in which the motor cortex is involved in motor control *via* neuronal preference for kinetics and kinematics, a dynamical system perspective emerging in the last decade views the motor cortex as a dynamical machine that generates motor commands by autonomous temporal evolution. In this review, we first look back at the history of the representational and dynamical perspectives and discuss their explanatory power and controversy from both empirical and computational points of view. Here, we aim to reconcile the above perspectives, and evaluate their theoretical impact, future direction, and potential applications in brain-machine interfaces.

## Introduction

The ultimate purpose of the nervous system is to produce appropriate action, and the motor cortex has long been thought to play a crucial role in planning and generating movement. Since the motor cortex was identified by Fritsch and Hitzig through surface electrical stimulation in the 1870s, several dogmas have been proposed to describe how it controls our musculoskeletal system. Anatomically, the motor cortex innervates the motoneuron pool in the spinal cord to drive skeletal muscles, and its neurons are clustered in accordance with the musculature following a somatotopic map [1]. Neurophysiological studies in non-human primates revealed that neuronal activity in the motor cortex is tuned to single-joint movements [2] and isometric force [3]. Since the 1980s, further studies of whole-arm movements have demonstrated that activity in the motor cortex represents a variety of motor parameters, such as direction [4], speed [5, 6], trajectory [7], and posture [8, 9].

Although the above representational perspective that directly maps neuronal activity to movement parameters is straightforward and has fostered brain-machine interfaces (BMIs), it still cannot explain the heterogeneous, complex, and time-varying firing patterns exhibited by many neurons in the motor cortex [10]. Since the 2000s, advances in neural interface and data science have enabled us to record and analyze large-scale neural signals. Recent studies have progressed from analyzing individual neurons to a systems approach, to the collective operation of neuronal populations. In line with this progression, Shenoy and colleagues proposed a dynamical system perspective [11, 12] which views the motor cortex as a dynamical machine that autonomously evolves during execution to issue descending motor commands. Based on the evaluation of neural data with a complex spatiotemporal structure, the dynamical system perspective has provided a deeper insight into high-dimensional neural trajectories in relation to motor planning and execution.

In this review, we not only summarize the paradigm shift from parametric representation to a dynamical system perspective, but also aim to reconcile these two viewpoints, which seem contradictory at first glance. Moreover, we present a global view of the integration of neural dynamics in the motor cortex with empirical studies on cortico-subcortical motor circuitry, theoretical work on the internal model, and BMI applications.

## Representation of Movement Parameters in the Motor Cortex

**Fig. 1:**
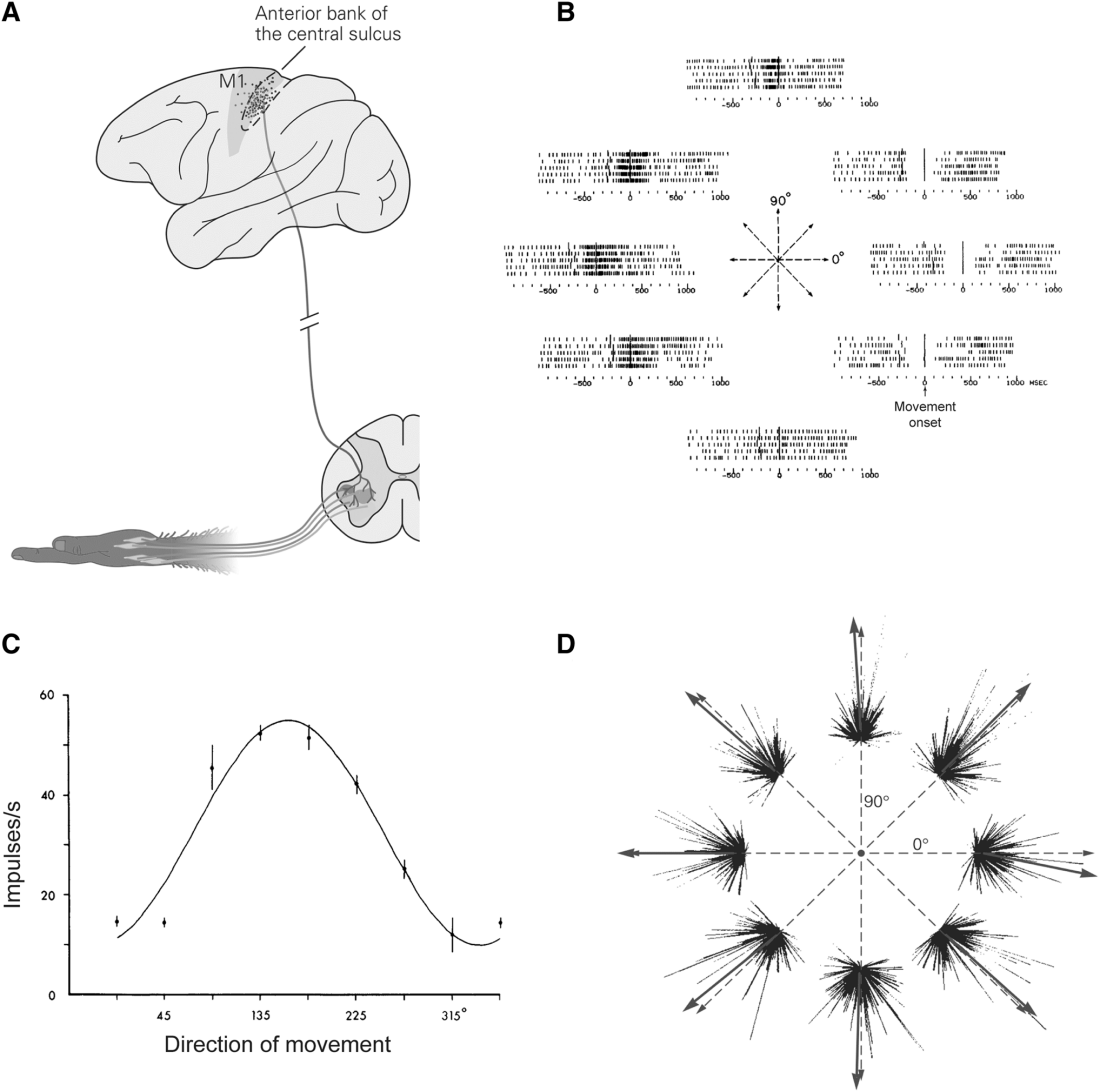
Illustration of the representational perspective. **A** M1 neurons mainly project to interneurons in the spinal cord *via* the corticospinal tract. Specifically, in primates, the corticomotoneurons are mainly located in the M1 sulcus, project monosynaptically to the motoneuron pool in the ventral horn. **B** Neuronal activity varies when monkeys push the manipulandum in different directions in the center-out task, which indicates selectivity for movement direction. **C** Firing rate of M1 neurons can be regressed with movement directions as a cosine function. The direction with the highest firing rate is called the preferred direction (PD). **D** Single neurons are represented as vectors with PDs whose length is the firing rate. The summation of these vectors is congruent with the actual movement direction. **A** and **D** adapted from Principles of Neural Science, fifth edition, 2013: 835–864 [128] with permission from McGraw Hill; **B** and **C** adapted with permission from Georgopoulos *et al*., 1982 [4].

A fundamental doctrine of neuroscience is that brain structure and connectivity determine functionality. A central goal of neurophysiology has long been to determine where the function is implemented, and how the information is represented in various brain areas. Following this principle, Fritsch and Hitzig identified the motor cortex by determining an area of the cerebral cortex from which movements were evoked by the application of electrical stimulation (for review, see [13]). Now we know that both the primary motor cortex (M1) and premotor cortex send descending projections to the spinal cord *via* the corticospinal (CS) tract [14, 15]. The descending CS influence on muscles is indirectly mediated by spinal interneuronal circuits (Fig. 1A). From monkeys to apes to humans, an increasing number of CS axons directly innervate motoneurons in the spinal ventral horn [15, 16], forming the cortico-motoneuronal projection. These anatomical findings provide a solid support for a causal role for the motor cortex in muscle control. Moreover, the motor cortex also receives projections from the visual and somatosensory cortices [17], which provide information about the external environment and internal body status essential for motor control. Taken together, the motor cortex receives information about sensory input, motor intention, decisions, and body status, and generates motor commands that descend to the spinal cord and other subcortical areas [18]. Beyond the corticocortical and corticospinal connections, the basal ganglia-thalamocortical circuitry and corticocerebellar circuits also play important roles in motor initiation, termination, sensorimotor representation, and motor learning [19, 20]. Their outputs are delivered to the motor areas through the ventrolateral thalamus, while the motor areas project to the basal ganglia *via* the striatum, and to the cerebellum *via* the pontine nuclei [19, 21, 22]. Nonetheless, how the motor program is accomplished and its underlying computational mechanisms are still elusive.

Inspired by the visual system, where neuronal responses encode the properties of visual stimuli, neuroscientists initially investigated the motor cortex by determining how its activity represents motor variables. Influenced by the somatotopic anatomical organization of the motor cortex, Evarts utilized single-joint movements to determine how M1 activity varies with certain joint parameters, such as force, joint angle, and speed [2, 23]. Fetz and colleagues [3] simultaneously recorded electromyography (EMG) and neuronal activity in the motor cortex, and found that the facilitation of specific muscles can be induced by subthreshold intracortical microstimulation (ICMS). These studies suggested that M1 neurons encode detailed information regarding intrinsic skeletomuscular parameters and help drive the effectors to accomplish the desired movement. For whole-arm reaching movements, neural activity in M1 is tuned to the directions of the endpoint movements (Fig. 1B) [4], and the firing rate (or discharge, *D*) is related to movement direction *via* a cosine function (Fig. 1C):

$$D = b + a{\text{cos }}(\theta - {\text{PD}})$$

where *a* is modulation depth,* b* is baseline, *θ* is the movement direction, and PD is the preferred direction. Single-neuron activity in the motor cortex can be represented as a vector component projecting to the neuronal PD in accordance with the current firing rate, though the PDs might shift during motor adaptation [24, 25]. If a large population of neurons is recorded (Fig. 1D), a population vector algorithm [26, 27] is applied for BMIs [28] by using neuronal PDs and their instantaneous firing rates to decode the actual movement direction. First, the weight of each neuron in a time bin is calculated as the baseline-subtracted firing rate. Then, the resulting vector sum of all neurons’ weights, multiplied by the unit vector along their PDs, is defined as the instantaneous population vector, which closely points to the actual movement direction. The whole-arm reach movement seems a good task for examining the correlation between high-level extrinsic information and neuronal activity in the motor cortex.

Due to the degrees of freedom problem in multi-joint movements, it is easier to study motor planning at the kinematic level, and directional tuning becomes a prominent feature for evaluating the impacts of other factors. Hence, it is unclear how M1 neurons represent high-level parameters like hand path. Because the high-level parameters are usually correlated with low-level muscle responses, a substantial effort has been made to dissociate them. Kakei *et al*. trained monkeys to perform step-tracking movements while gripping a handle with three different postures [29] to distinguish extrinsic (movement direction) from intrinsic (joint/muscle contraction) coordinates. About half of their recorded neurons displayed a stable PD in the various intrinsic coordinates, suggesting that both “muscles” and “movements” are equally represented in M1. However, for most neurons the load applied during movement induced PD changes in M1 [30] within different 3D workspaces [31], which might have resulted from the task involving different covariates. Even for unconstrained arm movements, directional tuning is also time-varying and segmented into two or three tuning components, and varies with other parameters [10, 32].

Hatsopoulos argued that single neurons are tuned to a direction at both lead and lag times, thus resulting in temporally-evolving movement trajectories, rather than simply instantaneous movement parameters [7]. In this view, the PD shift in the center-out task could be induced by the mismatch between the preferred trajectory and the constant target directions.

In principle, the motor cortex performs not as a parametric representation to describe movement but as a repertoire to produce it. Soon after Fritsch and Hitzig found that brief electrical stimuli evoked twitches, Ferrier showed that longer electrical stimulation evoked complex movements [33, 34]. Graziano and coworkers refined this experiment using a behaviorally relevant duration of ICMS, and found that the evoked behaviors of monkeys were complex, coordinated, and “purposive” [35, 36]. Moreover, the stimulation sites in the macaque motor cortex are clustered according to categories of evoked actions, so it is difficult to build an explanatory model following the repertoire hypothesis. The potential dimensionality of movement categories makes it a non-deterministic polynomial problem, and the continuity and flexibility of natural movements are challenging for data collection and analysis.

An alternative approach to refining the representational model is to introduce more parameters. For instance, movement speed is also conveyed in the motor cortex, and a gain-offset modulation model can fit this correlation well [5, 37]. Like the aforementioned posture effect, the PD shift was believed to be the result of sensorimotor transformations [38]; this is accomplished by posture-related gain modulation in a recurrent network of extrinsic-like units with different preferences [39–41].

## Heterogeneity and Complexity of Firing Patterns in the Motor Cortex

The representational perspective is more focused on the “encoding-decoding” problem. Especially for movement kinematics and kinetics, it attempts to build the representational function in the generalized form:

$${r}_{n}(t - \tau_{n} ) = f_{n} [param_{{1}} (t),param_{{2}} (t),param_{{3}} (t) \ldots ]$$

where neuronal activity *r*_*n*_ is jointly tuned to movement parameters *param*_*i*_ and time lag τ_*n*_ is used to cover the neuron-specific latency between cortical activity and parameters; neuronal conjunctive tuning to several variables is called mixed selectivity. If the tuning functions for each parameter are independent, *f*_*n*_ is a linear function [5, 7, 12, 28].

**Fig. 2:**
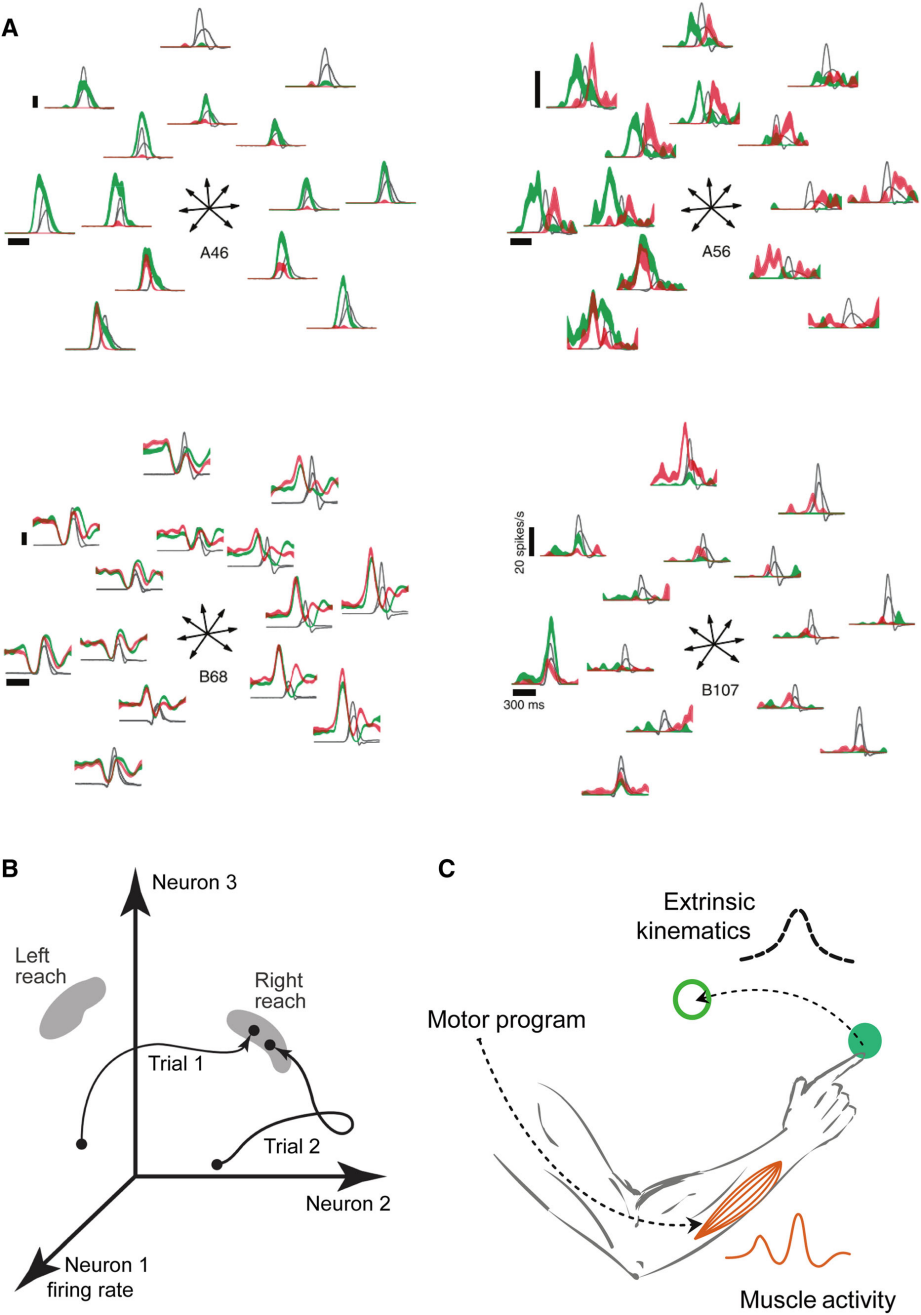
Illustration of the dynamical systems perspective. **A** In this task, the monkey performs a center-out reach with two distances and two hand speeds (gray, averaged hand velocity; red and green, mean firing rates fast and slow reaches, respectively). For neuron A46, tuning width varies with hand speed; for A56, the neural response leads the velocity profile in slow reach, while lagging in some directions of fast reach; B68 shows a multiphasic pattern with a different directional preference between preparatory and execution periods; and B107 presents a neuron that is exited only for long-distance reaches with speed-varied PD (adapted with permission from Churchland and Shenoy, 2007 [10]). **B** Neurons form a high-dimensional state space where the brain state evolves towards the optimal preparatory region to accomplish motor preparation (adapted with permission from Churchland *et al*., 2006 [80]). **C** The efferent descending motor program from neural dynamics leads the temporal sequence of muscle activation and holistic movement.

Another problem is the heterogeneity of neuronal activity in the motor cortex. As noted above, further studies on the tuning properties revealed that the temporal pattern does not always follow the representational model. In contrast to responses in the visual system that are triggered by the stimulus and maintained with stable preference, many neurons in the motor cortex exhibit ramping activity before movement onset, so-called preparatory activity, and a rapid bell-shaped peri-movement activity, although some neurons exhibit execution activity only, and others may show opposite tuning between preparation and execution. In addition, movement speed might not only reflect the change of firing rate and PD, but also the temporal relation between the neuronal response and the movement (Fig. 2A, [10, 32]). Even though some of the above findings might be explained by better behavioral measurements and by introducing more parameters into the representational model, they cast doubt on the model’s reliability. Following the doctrine that individual neurons are the basic computational units that represent information during each epoch in motor generation, such as the translation from extrinsic to intrinsic, from high-level to low-level, or formation and adjustment of the internal model, every stage and intermediate variable should be represented by the corresponding neuron, and all neurons together should formulate the movement command like the population vector. However, to compensate for the heterogeneous and time-varying tuning properties in the framework of representational perspective, nonlinear functions, and temporal profiles must be introduced, leading to increasingly complicated descriptive models without generalization.

## Neural Population Dynamics in the Motor Cortex

Interestingly, although single neurons show great heterogeneity, the linear decoding algorithms maintain stability and efficiency, indicating a robust linear readout at the population level, although there is a heterogeneous nonlinear response at the single-neuron level [26, 42, 43]. Several hypotheses have been proposed to explain this phenomenon. The reliability of linear decoding algorithms such as population vectors may be due to the existence of a large population of neurons related to hand directions with uniformly distributed PDs [44]. The mixed selectivity produces high-dimensional neural representations, and enables a linear readout for every task-relevant parameter [45, 46]. In a neural network, it is granted that the neuronal connectivity preserves a coordinating functional organization with intrinsic dynamical evolution. Noise correlation and preference distribution somehow may reflect this dynamic procedure [47–49]. This neural constraint by connectivity is also related to population plasticity in motor learning [50–53]. On the other hand, both kinematic and kinetic spaces cannot explain as much neural population variance as the peri-movement space [11], indicating that neural activity contains task-irrelevant elements. Churchland *et al*. tried to interpret the neural population in the motor cortex as a dynamical system and applied jointed principal component analysis (jPCA) to extract components of evolution that form a temporally oscillating structure. Because the oscillation emerged from not only a rhythmic movement, but also from a single reach, they claimed the rotations of the populational state are a prominent feature of the motor cortex. This simple and consistent feature challenges the framework of representational perspective by emphasizing the population state evolution which can be described with ordinary differential equations as the dynamical system (Fig. 2B, C) [54]. It has been recently revealed that the “hand knob” area in the human premotor cortex is indeed tuned to the entire body; following the dynamical system view, its control is supposed to be accomplished through limb-specific parts and general movement dynamics rather than the motor homunculus [55].

However, Lebedev *et al*. argued that the oscillation structure is only a byproduct of jPCA, by which any neural population with temporal shifts of individual neurons’ firing rates, and a condition-specific temporal sequence, would result in such an oscillation structure. Therefore, it is an exaggeration to claim that the structure is related to “an unexpected yet surprisingly simple structure in the population response” which “explains many of the confusing features of individual neural responses.” [56]. Later studies further addressed the “epiphenomenon” problem and tended to agree with both of the opinions that the oscillation structure is a byproduct, while the population dynamics during reach is better explained by a dynamical system than representational framework [57]. The significant difference is possibly embedded in the covariance across time, neurons, and conditions [58].

Although the dynamical perspective inspired a new direction for understanding population activity and improved comprehension of the motor cortex, it seems to dwell in the qualitative description and visualization of high-dimensional data, but to lack a tight link to the behavior as the representational perspective. Understanding the encoding of the population dynamics, and the triggering and control of temporal evolution will require further quantitative approaches.

## Dimensionality Reduction and Neural Manifold

A variety of dimensionality reduction methods have emerged, enabling selective extraction of information from high-dimensional neural data. The resulting principal components are believed to be the epitome of complex neuronal activity, as they are chosen to preserve or highlight some instructive characteristics in the data [59].

In practice, components acquired with different dimensionality reduction methods reveal different structural features of the data. For example, the most widely used method, principal component analysis (PCA) can identify components capturing the largest variance, and meanwhile orthogonal to each other, making it efficient for separating the dominant dynamics linearly. In contrast, factor analysis (FA) leads to components regarding shared variance. In addition to these two methods based on the covariance of trial-averaged neural data, there are also unsupervised methods to depict the temporal dynamics of single-trial population activity in time-series data, such as hidden Markov models (HMM), Gaussian process factor analysis (GPFA), latent linear dynamical systems (LDS), and latent nonlinear dynamical systems (NLDS). Methods to preserve dependent variables have been developed as well: linear discrimination analysis (LDA) can maximize cross-group variance compared to the within-group variance if given the number of separate groups, while demixed PCA (dPCA) gives principal components according to discrete task-relevant parameters and their possible combinations (for review, see [59–61]).

Dimensionality reduction facilitates observation and understanding by realizing the visualization of data in a low-dimensional space, for the time-varying neural activity can be represented as continuous neural trajectories or the instantaneous neural states in the space defined by principal components. For instance, the fact that the largest component detected by dPCA was nearly condition-invariant, but time-varying, suggests the apportion for neural encoding in the motor cortex [62]. In addition, the clustering and separating of neural states corresponding to different task variables in a low-dimensional space implicate distinct neural encoding rules, and thus help to distinguish different cortical regions [63].

**Fig. 3:**
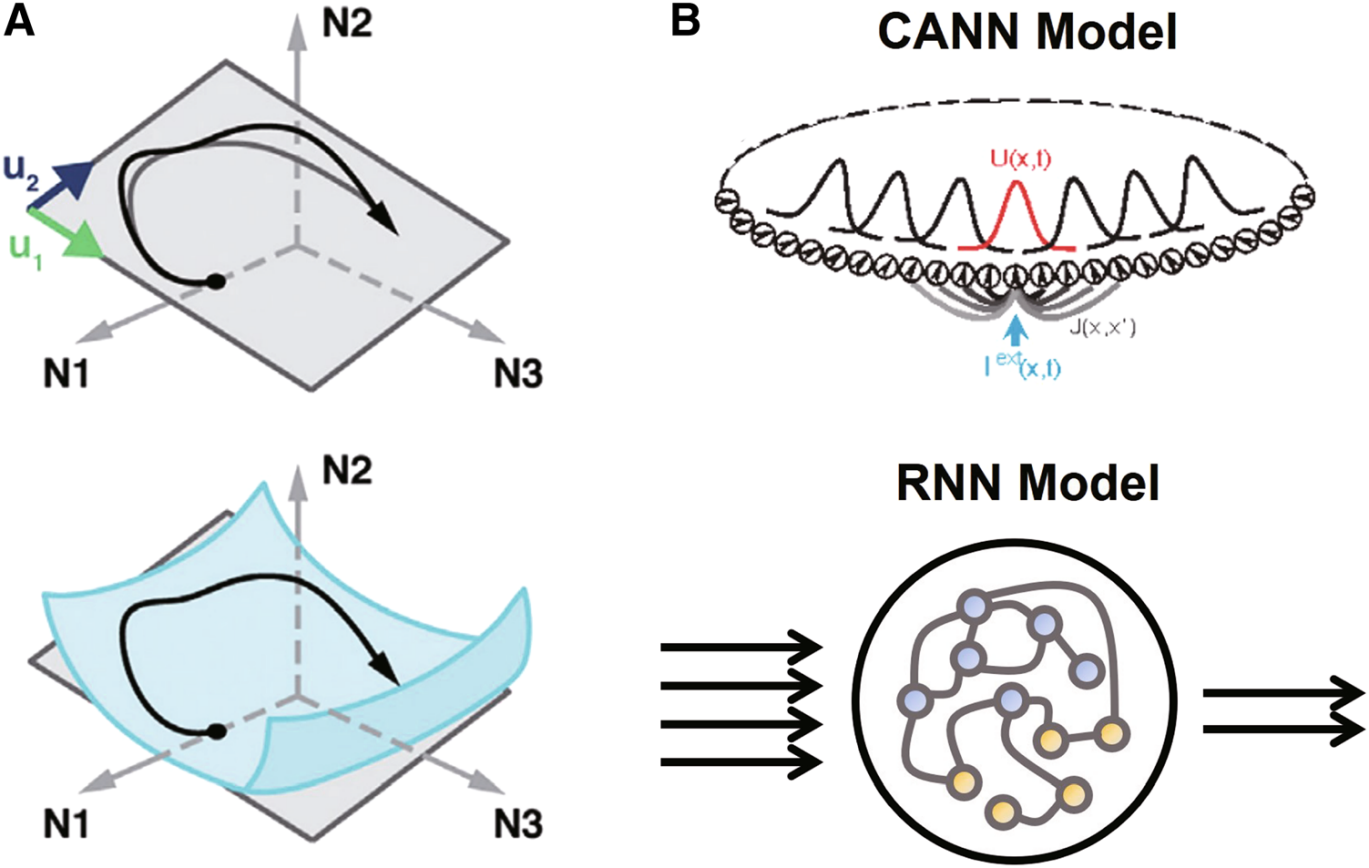
Illustration of neural manifolds and two kinds of recurrent network models. **A** Neural manifolds. The activity of three neurons (N1, N2, and N3) can be captured by a manifold spanned by two neural modes (${u}_{1}$ and ${u}_{2}$, as basic vectors). As a specific space defined by latent and shared neural activity patterns, a neural manifold can be approached by linearization despite its curvature in higher dimensions (adapted with permission from Gallego *et al.,* 2017 [64]). It is implied that the manifold underlies movement preparation and generation, because the necessary neural activity is expected to evolve on it. **B** Diagram of Continuous Attractor Neural Networks (CANNs). A CANN receives the external input $I^{ext} \left( {x, t} \right)$ and the synaptic input $U\left( {x, t} \right)$ at time $t$ for neurons with preferred stimulus at $x$. All the model neurons are connected with each other, in a way that the difference between their preference for a stimulus determines the strength of connectivity, $J\left( {x, x^{\prime} } \right)$ denoting the interaction from the neuron at $x$ to the neuron at $x^{\prime}$, (adapted with permission from Wu S *et al.,* 2016 [88]). Therefore, this kind of recurrent network is highly structural and analytical. **C** Diagram of Recurrent Neural Networks (RNNs). The RNNs in modeling motor control now are usually based on dynamic nodes. That is, the neural nodes evolve following a differential equation, rather than being filtered by simple activation functions. The yellow dots denote the nodes with only inhibitory (negative) connections while the purple dots denote the others, as a possibility. The inputs for these networks can be external signals in step form, while the outputs so far have been EMG, velocity, and hand trajectories.

More importantly, given that they are not actual neuronal activities, what is the connotation or essence of these principal components? It has been speculated that these components are key elements underlying behavior-relevant firing patterns that generate motor commands. This idea is embodied in a “manifold” theory, in which a manifold appears as a stable space restricted by some potent neural activity patterns called “neural modes” (Fig. 3A) [64]. It has been reported that a consistent neural manifold serves as the base for multiple motor behaviors [65], implying the possibility of a few basic sets of neural modes shared by the neural population.

Moreover, the finding that long-term learning would induce novel neural response patterns [52], shows the flexibility of such movement-relevant manifolds. On the other hand, the perturbation on manifold or off manifold are both favored to correlational studies, as they raise mechanistic hypotheses related to behavior [66].

Furthermore, principal components suggest functional partition in high-dimensional neural dynamics. It has been reported that subspaces captured by low-dimensional neural responses in different epochs, like preparatory and movement subspaces, are nearly orthogonal to each other, demonstrating their distinguishing functions [67, 68]. Beyond these period-relevant subspaces, a new method called preferential subspace identification (PSID) has lately been developed to model the neural dynamics relevant to behavior [69]. The transitions between subspaces and their relationships are expected to promote the understanding of the neural mechanism for motor control.

## Computational Models of Neural Dynamics for Movement Generation

With the observation of common tracks for neural trajectories from single trials in the same task, the temporal evolution of the neural population is deemed to generate movement itself, other than its parametric representation. In this sense, the framework of neural dynamics, which is a subject that studies those systems evolving with time [70], has been introduced.

In fact, it is not novel to view the cortex as a dynamical system: Decades ago the dynamical systems perspective nourished central pattern generator theories for the spinal cord and an equilibrium point hypothesis for motor control [71]. However, just as the analogy between a dynamical system and mind was controversial in the 1990s [72, 73], it is intriguing today to consider the motor cortex from a dynamical system perspective.

An approach to casting the motor cortex as a dynamical system is to regard the nervous system as a machine that generates an appropriate neural response pattern to trigger the holistic movement [12]. In this framework, time-varying neuronal activities contribute to motor control as drivers, and the temporal evolution can be independent of the kinematic or kinetic parameters. In general, the temporal pattern of neuronal activities *r*(*t*) can be described with a differential equation,

$$\tau r(t)=h[r(t)] +u(t)$$

where the temporal derivative of neural activity $\dot{r}$, is modulated by a time constant τ and impacted by the local interactions in the motor cortex *h*( ) and the input from other brain areas *u*(*t*) $s r_(t) = h[r(t)] + u(t)$ [12]. In such a system, the initial states, the synaptic inputs due to connectivity among local circuits, the external inputs, and even the time constant, all have a considerable influence on neural activities.

Nevertheless, it has not been without confusion to verify the existence of such a dynamic system. For this purpose, surrogate datasets designed randomly, but sharing certain features of the original data were built with the “corrected Fisher randomization” (CFR) and “tensor maximum entropy” (TME) methods. As a result, preserved features alone cannot reproduce the dynamical structure in real neural data [58]. Thus, although this did not directly unveil the neural dynamical system, it indicated that the neural dynamics changed with an intrinsic logic.

To uncover a neural dynamical system, one cannot avoid depicting it. For this purpose, the neural trajectories now take on new values, since they not only exhibit the real-time neural states, but also indicate trends in the phase space. Measurements of condition-varying trajectories thus offer insights into neural mechanisms. The length, speed, and curvature of single trajectories, along with the angle between them, can be calculated to test hypotheses in a differential geometric way [74, 75]. While neural trajectories from data show the actual situations, “fixed points”, as one of the most salient features of phase portraits, can be even more significant because of their ability to predict situations starting from new initial states [70]. In neuroscience, stable fixed points or attractors, corresponding to steady states or equilibria of the system, can be regarded as stable response patterns such as memory [76, 77] or appropriate states necessary for movement [78].

Finding specific neural states that reflect the dynamical system is promising, but just the beginning. It is more important, but difficult, to figure out how the system is related to the behavior. In other words, how to explain the neural mechanism with the structure of the proposed dynamical system. It has been shown that one-dimensional dynamics are enough to model the transition from spontaneous activity to delay activity in the macaque lateral intraparietal area for spatial attention diversion [79], but things get more complicated for motor control. For a specific example of arm movement, the preparatory activity has been proposed to act as the initial state of a dynamical system for action [11]. In neural space, the population dynamical states converge to “a relatively tight set” after the appearance of targets (Fig. 2B). This set, called the “optimal subspace”, is supposed to benefit evolution to the desired motor command. According to the optimal subspace hypothesis, the goal of motor preparation is to set the population dynamical state into this optimal preparatory subspace [12, 80]. In fact, it is impractical to build a unified dynamical system for the entire movement generation process, for it has been demonstrated that different motor areas contain distinct neural dynamics [81]. Like the optimal subspace hypothesis, period- or location-specific dynamical systems may have more practical value at present.

Meanwhile, modeling efforts under the dynamical system perspective have been emerging [82–85]. Take the classical model of two-interval discrimination as an example. It is a simple mutual-inhibition network model that captures all task phases within a single framework. In this model, the population of neurons is simplified as an excitatory and an inhibitory node. Then the phase-plane plot of input/output functions of these two nodes is sufficient to reveal the dynamics in each phase, including the shift of stable fixed points as well as the appearance and disappearance of line attractors [82].

While discrete attractor dynamics have recently been shown to support short-term memory associated with motor planning in mice [86], continuous attractor dynamics seem to have entered the field earlier. An early form of continuous attractor neural network (CANN) was a neural field consisting of several types of neurons as homogeneous subnets. This network could obtain equilibrium solutions without input, and react to a stimulus of stationary patterns [87]. Now CANNs (Fig. 3B, top) can be regarded as a kind of recurrent network adept at information representation, for stimuli can be encoded as their stable activity patterns (attractors). The translation-invariant connection determines this kind of network and becomes the most prominent feature [88]. In the field of motor control, CANNs have been applied to explain the encoding of continuous changing direction [89] and anticipative tracking [90].

Recurrent network models are established under the dynamical system perspective as well. Recurrent neural networks (RNNs, Fig. 3B lower), whose neurons evolve according to a group of ordinary differential equations, can generate desired EMG signal patterns after training. Moreover, the dynamics of either single neurons or the population of the model are comparable to real data [91]. In many applications, RNNs show eminent flexibility that is attributable to the adjustable connection structure [57, 92–96]. This plasticity, along with their temporal extensibility, makes RNNs the first choice for studies on the emergent property and behavior-relevant neuronal activities. Nonetheless, RNNs with fixed structural connectivity have recently been built. In an excitation-inhibition balanced recurrent network, which can generate complex movements, the neural dynamics also largely agree with experimental findings. The optimization for the stability of the connectivity in this network was guided by dynamics theory [97]. Except for being applied to build network models, mathematical knowledge and techniques in dynamics have also been introduced to open the “black box” of high-dimensional RNNs. In a theoretical study, the effect of linearization in realms of phase space around fixed points or points with very slow movements was explored. In the example cases provided, the mechanisms of networks could be deduced from linearized dynamics around these important points [98].

## Perspectives

Emerging as a new framework for understanding the neural basis of motor control, the dynamical systems perspective indeed is a complement or extension of the representational perspective, rather than a firm refutation. It emphasizes that the autonomous dynamical evolution is predominately determined by preparatory activity, consistent with the central concept in the prevalent theory of motor control, the internal model [99, 100]. Numerous behavioral and computational studies suggest that the motor program is inversely preplanned (inverse model) based on the forward model of future states, rather than adjusted online relying on continuous sensorimotor transformation during execution [101, 102]. From our point of view, dynamical evolution from initial neural states set by preparatory activity provides a plausible neural mechanism underpinning the internal model.

**Fig. 4:**
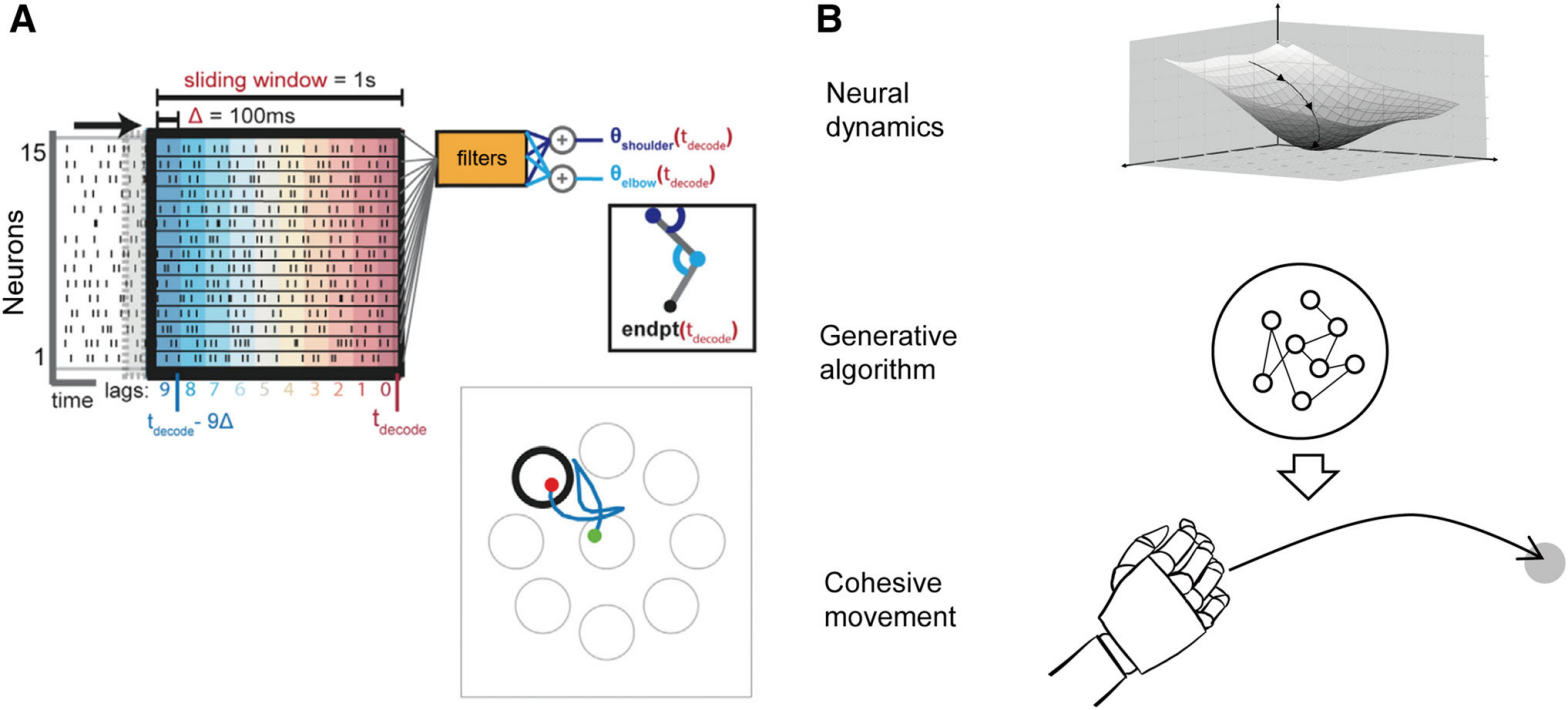
Decoding frameworks of BMIs based on representational (**A**) and dynamical systems (**B**) perspectives. **A** Neural activities within a certain sliding window ahead of the decode time ${t}_{decode}$ are binned with 100 ms to form a high-dimensional matrix. BMIs based on representational perspectives usually use fixed decoders like the Wiener filter to map high-dimensional neural activities into low-dimensional control signals. From the start (green point) to the end (red point), the endpoint (endpt) trajectory (blue line) is segmented due to feedback adjustment (modified with permission from Athalye *et al*., 2017 [113]). **B** Schematic for BMIs from the dynamical systems perspective. This regards the motor cortex as a machine to generate proper neural activity patterns for the desired movement, and thus the movement can be implemented once the mapping between neural dynamics and concrete muscle activity is defined. In this situation, it is essential to first figure out stable neural states, for they function as attractors, and then design reliable generative algorithms (e.g. RNNs) for efficient neuron-to-muscle signal transition.

In principle, if the neural dynamical machine in the motor cortex autonomously generates motor commands, a cohesive motor program could be decoded from preparatory activity to rapidly drive an external actuator to implement BMIs. Although some models aimed to link neural dynamics and the muscle/arm [91, 97, 103], remarkable advances in BMIs over the past two decades largely relied on a representational perspective [104–110]. In the current BMI framework, a decoder first is trained to find a parametric mapping between recorded neural activity and movement covariates, and then it continuously converts neural activity to control variables guiding external objects (see review, [111]). For this representative mapping, the population vector algorithm, as noted before, makes use of clear analytical relations which are intuitive and interpretable. In fact, the essence of decoders based on representational perspectives does not go beyond the population vector algorithm. Putting aside cosine tuning and Cartesian coordination, Sanger showed that the population vector can be found with the simple assumptions that neurons respond to behavior in a predictable way, and that neuronal preferences are approximately uniformly distributed in task space [112]. However, this frame is static, essentially depending on the previously-recorded fluctuating neural responses. Consequentially, movement of the actuator requires the continuous adjustment of brain-controlled signals without prior trajectory formation, unlike naturalistic movements planned in a feedforward manner, leading to unsatisfactory performance of BMIs for viable clinical applicability in terms of motion speed and smoothness (Fig. 4A, [113]).

In contrast to discriminative decoding algorithms fostered by representational perspectives that continuously translate neural signals into movement parameters, the decoders inspired by the dynamical systems perspective should be temporally generative to yield an integrative control program based on preparatory activity (Fig. 4B). Ideally, clinically feasible BMIs should be able to interact with dynamic environments in realtime, demanding a feedforward controller to produce ballistic movements [114–116]. Recently, in nonhuman primates, we tested a BMI with a generative model to intercept moving objects indicating potential advantages of feedforward control in dynamic BMI design for more biomimetic and flexible neuroprosthetics [117, 118].

So far, BMIs have distinguished themselves from a static decoder in various aspects. The good performance of current static decoders based on discriminative models may be due to neural redundancy and low task dimensionality [46, 64, 119, 120]. However, considering the limited, biased, and unstable sampling of daily neural recordings, a generative model would be preferred for a more naturalistic prosthesis.

Moreover, while a static decoder is not suitable for dynamic sensorimotor contingencies [121], novel BMIs based on neural dynamical systems enable the sophisticated integration of feedforward control and multi-modal feedback (e.g., *via* ICMS) [116, 122]. While the external device is controlled by only cortical signals, the control and feedback of brain-controlled neuroprosthetics are different from natural movements, leading to novel neural responses [51–53, 123], suggesting an inherent difference between BMI control and neural decoding.

Furthermore, an optimistic dynamical perspective emphasizes the importance of initial state and temporal dynamics within the cortex, which subsequently triggers the detailed control program in subcortical and spinal circuitry [12, 124, 125], demanding hierarchical decoding algorithms for next-generation BMI control.

From our viewpoint, neural population dynamics and single-neuron characteristics complement each other. Neural population dynamics rely on the coordinated tuning of individual neurons, whereas single neurons must be spatiotemporally orchestrated to generate motor commands. On the other hand, the parametric representation and the dynamical systems perspectives are two sides of a coin. It is fair to suggest that the representation perspective asks, “what motor parameters are involved?”, while the dynamical system perspective focuses on “how does function evolve in time?”. Since the dynamical states can be representational [126], it is reasonable to hope that these two perspectives can be incorporated into one framework, though this will demand great effort. In the future, it will be helpful to quantitatively link neural population dynamics and holistic physical movement, as well as to identify the recurrent neural circuitry underlying dynamical rules and external triggers for the transition from preparation to execution [127]. Studies on the initial state, local dynamics, and external inputs of a dynamical system could provide inspiration. Nevertheless, it is still unclear if neural dynamics emerge from the motor cortex alone or a larger brain network. Thus, it is important to identify the specific roles of multiple brain areas in future studies.
